# Supplementary material for: A Novel Triple-Action Inhibitor Targeting B-Cell Receptor Signaling and BRD4 Demonstrates Preclinical Activity in Chronic Lymphocytic Leukemia
Source: Int J Mol Sci. 2022 Jun 16;23(12):6712. doi: 10.3390/ijms23126712 (PMC9224275; doi:10.3390/ijms23126712)
Supplement: Supplementary file 1 [file ijms-23-06712-s001.zip › ijms-1748030-supplementary.pdf]

Supplementary Materials

Supplementary Methods

Immunoblot analysis

Samples were lysed in RIPA buffer containing Sigma Protease and Phosphatase Inhibitor Cocktails and phenylmethyl sulfonyl fluoride (Sigma-Aldrich, Saint Louis, MO), and analyzed by sodium dodecyl sulfate polyacrylamide gel electrophoresis (SDS-PAGE). Gels were transferred onto nitrocellulose membranes using Bio-Rad's TransBlot Turbo Transfer System, probed with primary antibodies and HRP-conjugated secondary antibodies (Bio-Rad Laboratories, Hercules, CA). Blots were then visualized on Bio-Rad's ChemiDoc MP imager using SuperSignal West Pico PLUS Chemiluminescent Substrate or Femto Maximum Sensitivity Substrate (Thermo Fisher Scientific, Waltham, MA) according to the manufacturer's instructions. ImageJ software was used for densitometric band quantification [1].

Primary antibodies against phospho-BTK (Tyr223), BTK, phospho-PRAS40 (Thr246), PRAS40, phospho-ERK (Thr202/Tyr204), ERK, MYC, P21, and GAPDH were obtained from Cell Signaling Technologies (Danvers, MA). Anti-β-TUBULIN was ordered from Sigma-Aldrich and anti-HSP 90α/β was obtained from Santa Cruz Biotechnologies (Santa Cruz, CA).

Supplementary Data

Supplementary Tables

Table S1. Key characteristics of the malignant B-cell lines used in the study

Cell Line	Disease Type	Key Characteristics
MEC-1 [1]	CLL	Mutated IGHV, 17p deletion, complex karyotype [2]
MEC-2 [1]	CLL	Mutated IGHV, 17p deletion, complex karyotype
OSU-CLL[3]	CLL	Mutated IGHV, non-complex karyotype
HG-3 [4]	CLL	Un-mutated IGHV, non-complex karyotype
OCI-LY3 [5]	ABC-DLBCL	t(14;19)(q32;q13) IGH-SPIB, amplified BCL2<br>MYD88 mut, CARD11 mut
SU-DHL-6 [6]	GC-DLBCL	t(14;18)(q32;q21), IGH-BCL2, rearranged BCL6 gene, EZH2 mut

1 MEC-1 and MEC-2 cell lines represent spontaneous outgrowths of CLL clones on two subsequent occasions (1 y apart) from a patient with mutated IGHV CLL and a complex karyotype with 17p deletion ($P53^{mutated}$). MEC-2 cells have evident prolymphocytic leukemia transformation [2,3].

2 Complex karyotype is defined as having ≥3 chromosomal aberrations.

3 The OSU-CLL cell line originated from a CLL patient with mutated IGHV and non-complex karyotype [4].

4 The HG-3 cell line originated from a CLL patient with un-mutated IGHV and demonstrates non-complex karyotype with biallelic 13q14 deletions [5].

5 The activated B-cell like DLBCL (ABC-DLBCL) cell line, OCI-LY3 carries cryptic t(14;19) with rearrangement of IGH and SPIB and contains copy number amplification of the BCL2 region [6-8]. OCI-LY3 cells harbor mutations (mut) in CARD11 and MYD88 (L265P) [9,10].

6 The germinal center-like DLBCL (GC-DLBCL) cell line, SU-DHL-6 carries t(14:18) effecting IGH-BCL2 fusion and has BCL6 gene rearrangement [7,8]. SU-DHL-6 cells harbor the EZH2 Y641N mutation [11].

Table S2. Characteristics of CLL patient samples used in the study

ID	Gender	Age	IGHV status	Treatment status	Karyotype	Figure(s)
6-0615	F	65	U	N	47,XX,+12(1)/46,XX(19)	3A, 3B (patient #1*)
6-0726	M	58	U	N	46,XY(23)/nonclonal(4)	3A, 3B (patient #2*)
12-1833	M	76	U	T	44,XY,t(1;19)(q21;p13.1),-4,der(8;18)(q10;10),-17,+der(?)t(?;4)(?;q12)[cp10]/44,sl,add(1)(p36.1),add(9)(q31)[cp4]/44,sl,t(1;22)(p32;p11.2),-der(8;18),+der(8;18)(q10;q10)t(?;8)(?;q24)t(?;4)(?;q12),+mar[cp3]/43-45,XY,-4,t(5;16)(q13;p13.3),del(7)(q32),-17,add(22)(p11.2),+der(?)t(?;4)(?;q12)[cp4]	3B (patient #3*), 4A
6-1127	F	76	U	N	46,XX,del(11)(q13q23)(6)/45,X,-X(3)/46,XX(10)/nonclonal	3A, 3B
16-2100	F	79	U	T	46,XY,der(1)(11qter->q13::?::1p13->1qter),del(7)(q32q36),-11,der(20)t(15;20)(q15;q13.3),+mar[3,one w/ nonclonal abnormalities]/ 46,sl,add(8)(p11.2)[16,one w/ nonclonal abnormalities]/non-clonal[1]	3B
18-4770	M	50	U	N	47,XY,+12,t(14;19)(q32.3;q13.2)[10]/46,XY[10]	3A, 3B
8-1148	F	57	U	N	46,XX(19)/nonclonal(1)	3A
10-0090	M	58	M	N	46,XY(29)/nonclonal(1)	3A
9-0520	M	47	U	N	46,XY[20]	3A, 4A
10-1323	M	55	U	N	46,XY,inv(1)(p31q32),del(6)(q16q22),del(11)(q22q23)[14]/46,XY[6]	3A
6-0921	F	68	NA	N	47,XX,+12(4)/46,XX(8)	4A
15-1600	M	94	M	T	47,XY,+12[9]/47,sl,del(10)(q24q26)[11]	4A
5-0996	F	62	U	N	47,XX,+X(3)/46,XX(27)	4A
10-1072	M	47	M	N	49,XY,+12,+18,+19[2]/49,idem,t(X;13)(p11.4;q14)[15]/46,XY[3]	4A
16-3489	M	76	U	T	47,XY,+12[20,two w/nonclonal abnormalities]	4A
9-1726	F	60	U	T	46,XX,del(11)(q21q23),inc[cp3]/46,XX[1]	4A
10-1385	M	45	U	N	47,XY,+12,t(14;19)(q32.3;q13.2)[4]/46,XY[15]/nonclonal[1]	4A

M (mutated IGHV mutational status); U (unmutated IGHV mutational status); T (treated patient); N (treatment-naïve patient); NA (information not available); * (representative immunoblots are shown in Fig. 3B)

Supplementary Figures

Supplementary Figure S1

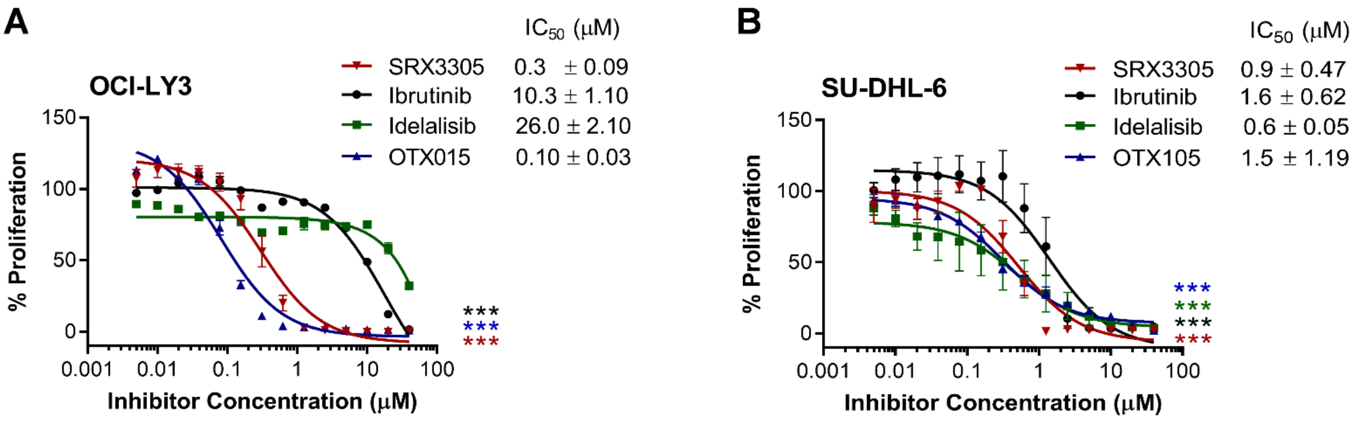

Figure S1. SRX3305 inhibits DLBCL proliferation. Two DLBCL cell lines, OCI-LY3 **(A)** and SU-DHL-6 cells **(B),** representative of ABC- and GC-DLBCL subtypes, respectively were treated with increasing doses of SRX3305 (BTK/PI3K/BET inhibitor), ibrutinib (BTK inhibitor), idelalisib (PI3K inhibitor) or OTX015 (BET inhibitor) for 72 h. Proliferation was assessed via MTS assay and results given as % proliferation normalized to vehicle. Error bars indicate SEM (n=3 independent experiments per cell line). Dose-dependent decrease in cell proliferation is observed with asterisks denoting significance (*** $P < 0.001$). IC_{50} values are noted for each inhibitor within the in-figure legends (mean ± SEM).

Supplementary Figure S2

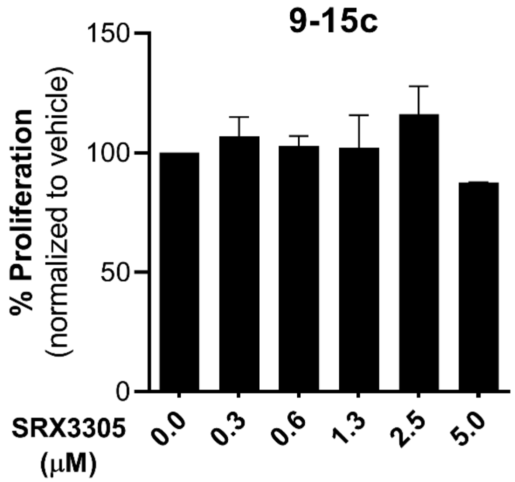

Figure S2. SRX3305 is not toxic to bystander stromal cells. Murine 9-15c bone marrow-derived stromal cell lines were treated for 72 h with DMSO vehicle or increasing concentrations of SRX3305 as indicated. MTS assay was done, and stromal cell proliferation is represented relative to vehicle control. Results are given as mean ± SEM (n=3 - 4 independent experiments).

Supplementary Figure S3

A

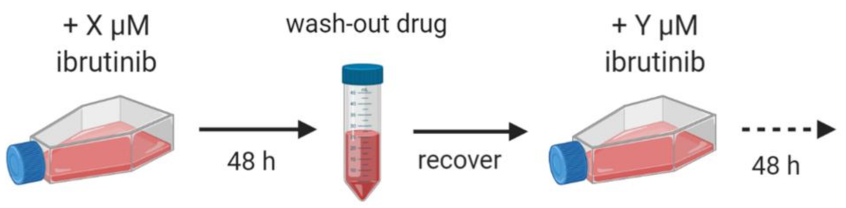

B

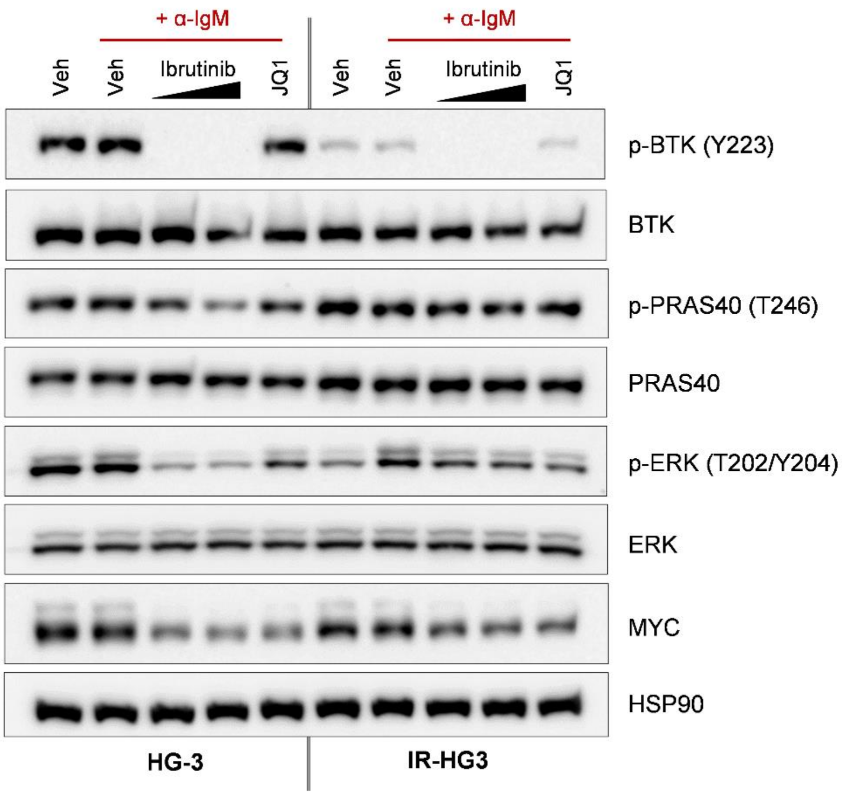

Figure S3. Generation of an ibrutinib-resistant HG-3 cell line model. (A) Diagram outlining steps taken to render HG-3 cell resistant to ibrutinib. HG-3 cells (1 e^6/mL) were treated with increasing doses of ibrutinib for 48 h at a time, then drug was washed out and the cells were left to recover to ≥ 80% viability before successive dosing (---►). Ibrutinib dose was not increased unless cells successfully recovered within 48 h. Once HG-3 cells remained viable in 25 μM ibrutinib, they were used for further analysis. **(B)** Whole cell lysates of parental (HG-3) and ibrutinib-resistant HG-3 (IR-HG3) cells were collected for immunoblot analysis of the indicated proteins (n=2 independent experiments per cell model). Representative immunoblots are shown.

References

1. Schneider CA, Rasband WS, Eliceiri KW. NIH Image to ImageJ: 25 years of image analysis. *Nat Methods.* 2012;9(7):671-675.10.1038/nmeth.2089
2. Rasul E, Salamon D, Nagy N, Leveau B, Banati F, Szenthe K, et al. The MEC1 and MEC2 lines represent two CLL subclones in different stages of progression towards prolymphocytic leukemia. *PLoS One.* 2014;9(8):e106008.10.1371/journal.pone.0106008
3. Stacchini A, Aragno M, Vallario A, Alfarano A, Circosta P, Gottardi D, et al. MEC1 and MEC2: two new cell lines derived from B-chronic lymphocytic leukaemia in prolymphocytoid transformation. *Leuk Res.* 1999;23(2):127-136.10.1016/s0145-2126(98)00154-4
4. Hertlein E, Beckwith KA, Lozanski G, Chen TL, Towns WH, Johnson AJ, et al. Characterization of a new chronic lymphocytic leukemia cell line for mechanistic in vitro and in vivo studies relevant to disease. PLoS One. 2013;8(10):e76607.10.1371/journal.pone.0076607
5. Rosen A, Bergh AC, Gogok P, Evaldsson C, Myhrinder AL, Hellqvist E, et al. Lymphoblastoid cell line with B1 cell characteristics established from a chronic lymphocytic leukemia clone by in vitro EBV infection. *Oncoimmunology.* 2012;1(1):18-27.10.4161/onci.1.1.18400
6. Mehra S, Messner H, Minden M, Chaganti RS. Molecular cytogenetic characterization of non-Hodgkin lymphoma cell lines. *Genes Chromosomes Cancer.* 2002;33(3):225-234.10.1002/gcc.10025
7. Quentmeier H, Pommerenke C, Dirks WG, Eberth S, Koeppel M, MacLeod RAF, et al. The LL-100 panel: 100 cell lines for blood cancer studies. *Sci Rep.* 2019;9(1):8218.10.1038/s41598-019-44491-x
8. Drexler HG, Quentmeier H. The LL-100 Cell Lines Panel: Tool for Molecular Leukemia-Lymphoma Research. *Int J Mol Sci.* 2020;21(16).10.3390/ijms21165800
9. Davis RE, Ngo VN, Lenz G, Tolar P, Young RM, Romesser PB, et al. Chronic active B-cell-receptor signalling in diffuse large B-cell lymphoma. *Nature.* 2010;463(7277):88-92.10.1038/nature08638
10. Ngo VN, Young RM, Schmitz R, Jhavar S, Xiao W, Lim KH, et al. Oncogenically active MYD88 mutations in human lymphoma. *Nature.* 2011;470(7332):115-119.10.1038/nature09671
11. Morin RD, Johnson NA, Severson TM, Mungall AJ, An J, Goya R, et al. Somatic mutations altering EZH2 (Tyr641) in follicular and diffuse large B-cell lymphomas of germinal-center origin. *Nat Genet.* 2010;42(2):181-185.10.1038/ng.518
